# Correlation between ERP and CSF biomarkers in Mild Cognitive Impairment and Dementia patients in a general neurological clinic population

**DOI:** 10.1002/alz.093657

**Published:** 2025-01-09

**Authors:** Igor Levy

**Affiliations:** ^1^ Neuroscience spine and associates, Naples, FL USA

## Abstract

**Background:**

We have recently reported on the use of Event‐Related Potentials (ERP) biomarkers to separate Amyloid PET positive vs Amyloid PET negative patients with mild cognitive impairment (MCI) and dementia. A large body of evidence shows that low levels of beta amyloid and increased tau in the CSF are highly correlated with Alzheimer’s pathology in the brain. We investigated the correlation between amyloid and tau in the CSF, and ERP responses in patients with MCI and dementia.

**Method:**

Patients with MCI and Dementia in a general neurological outpatient clinic underwent ERP testing. CSF analyses for Amyloid and TAU were also performed, and the results correlated with the output from the ERP test. When CSF studies were indeterminate, readings for brain amyloid brain load were obtained through PET analysis as part of the IDEAS (Imaging Dementia ‐Evidence for Amyloid Scanning) study when feasible

**Result:**

Data will be presented at the conference with correlation between ERP markers and CSF amyloid and tau levels in patients with MCI and dementia.

**Conclusion:**

Study results will provide valuable insights into the use of ERPs in a general neurological population with Alzheimer’s disease and cognitive impairment.

